# Supplementary material for: Strengthening Kangaroo Mother Care at a tertiary level hospital in Zambia: A prospective descriptive study
Source: PLoS One. 2022 Sep 1;17(9):e0272444. doi: 10.1371/journal.pone.0272444 (PMC9436113; doi:10.1371/journal.pone.0272444)
Supplement: S1 Appendix — (DOCX) [file pone.0272444.s001.docx]

**S1 Appendix: KMC PREDISCHARGE READINESS SCORING CHART**

| **KMC Daily score sheet** |  |  |  | Date | Date | Date |
| --- | --- | --- | --- | --- | --- | --- |
| **D.o.B** | **Name of Facility:** | | | Day 1 | Day2 | Day 3 |
| **File No.** | **Formular:** | | |  |  |  |
| **Evaluation** | **SCORES** | | | Kg | Kg | Kg |
|  | 0 | 1 | 2 |  |  |  |
| Social economic support | No help or support | Occasional  Help | Good  Support  System |  |  |  |
| Mother’s milk production  (Must score before discharge. NA  For formula feeding | Expresses0=10ml  Breast milk | Expresses  0-20ml  Breast milk | Expresses  20-30ml  Breast milk |  |  |  |
| Positioning & attaching  Of the baby on the breast ( not applicable for formula feeding) | Always needs  assistance | Occasionally  Needs  assistance | No assistance  Needed |  |  |  |
| Baby’s ability to suckle at the  Breast/cup feeds | Gets tired very  quickly | Gets tired infrequently | Takes all feeds well |  |  |  |
| Confidence in handling baby i.e feeding, cleaning baby, changing baby | Always needs assistance | Occasionally needs assistance | No assistance needed |  |  |  |
| Baby’s weight gain pay day  (must score 1 or 2 before discharge) | 0-10g | 10-20g | 20-30g |  |  |  |
| Confidence in administering  Vitamins &iron | No confidence | Some confidence | Fully confident |  |  |  |
| Knowledge of KMC | No knowledge | Some knowledge | knowledgeable |  |  |  |
| Acceptance & application of KMC | Does not accept or apply KMC | Partly accepts & applies KMC | Applies KMC without having to  be told |  |  |  |
| Confidence in caring baby at home | Does not feel sure or able | Feels slightly unsure & unable | Feels confident |  |  |  |
| **Total Scores per day** | | | |  |  |  |
| Read to discharge? | Yes No |  |  |  |  |  |
| **Breastfeeding:**  -if mother & baby score >19  **Formula feeding:**  -if mother &baby score >15 |  |  |  |  |  |  |
